# Better performance of Hartree–Fock over DFT: a quantum mechanical investigation on pyridinium benzimidazolate types of zwitterions in the light of localization/delocalization issues

**DOI:** 10.1007/s00894-023-05706-1

**Published:** 2023-09-13

**Authors:** Sanyasi Sitha

**Affiliations:** Department of Chemical Sciences, University of Johannesburg, Auckland Park, PO Box 524, Johannesburg, 2006 South Africa

**Keywords:** HF, DFT, Post-HF, Zwitterion, Dipole

## Abstract

**Context:**

With the advent of fast computing facilities, combined with rapid emerges of many new and intricate quantum mechanical functionals, computations with pure Hartree–Fock (***HF***) theory are now-a-days regarded as trivial or obsolete, or even considered as not reliable by many researchers. Consequently, current trends in computational chemistry show extensive use of ***post-HF*** theories for smaller molecular systems and various ***DFT*** methods for organic and inorganic chemistry related problems (larger molecules/systems). In this contribution, I have tried to show that sometimes, HF might be more suitable over DFT methodologies in addressing structure–property correlations. Molecules studied here were previously synthesized by Boyd in 1966 and important experimental data were produced by Alcalde and co-workers in 1987. Comparison of computed and experimental results clearly shows that HF method was more effective in reproducing the experimental data compared to especially the DFT methodologies. Reliability of HF method was further assured from the very similar results shown by the CCSD, CASSCF, CISD and QCISD methods. Current study also indicates that the localization issue associated with HF proved to be advantageous over delocalization issue of DFT based methodologies, in correctly describing the structure–property correlation for zwitterion systems.

**Methods:**

All computations were performed with Gaussian 09. A wide-range of quantum mechanical methodologies, *HF, B3LYP, CAM-B3LYP, BMK, B3PW91, TPSSh, LC-ωPBE, M06-2X, M06-HF, ωB97xD, MP2, CASSCF, CCSD, QCISD, CISD and semi-empirical methods like, Huckel, CNDO, AM1, PM3MM and PM6*, were used for investigations.

**Graphical abstract:**

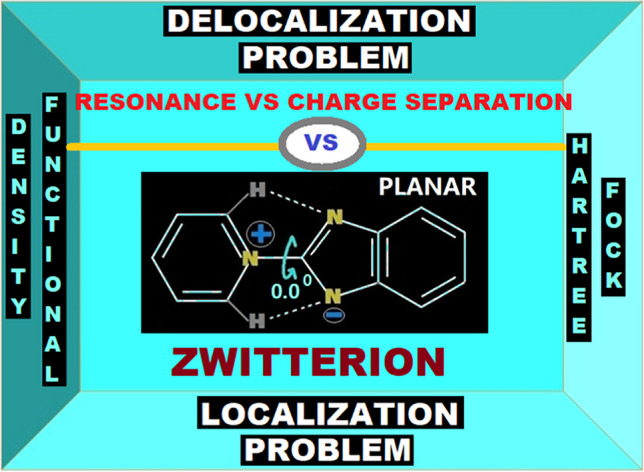

## Introduction

History of ‘Quantum Mechanics’ shows that, Hartree–Fock theory (HF) refers to the 1927’s original work of D. R. Hartree (published in January 1928). [1] Hartree’s work was presented to scientific community immediately after the 1926’s profound work of Erwin Schrödinger [2] (famously known as Schrödinger Equation, $i\hslash \frac{d}{dt}|\Psi (t)\rangle = \widehat{H}|\Psi (t)\rangle$). [3] In his work Hartree offered a systematic procedure, famously known as Self-Consistent Field (SCF) method (also recognised as Hartree’s Method), where he presented the solutions to determine energy and wave functions (approximate), for elementary quantum mechanical systems. [1] In 1928, J. C. Slater [4] and J. A. Gaunt [5] (both simultaneously and independently), demonstrated that the variational principle to a trial wave-function (an ansatz) can be considered as an appropriate theoretical basis for the SCF method. Then, in and around 1930, independently J. C. Slater [6] and V. A. Fock, [7, 8] applied the anti-symmetry to the electronic solutions. With the use of Slater determinant (of one-particle wavefunctions), [9] which was known to have the essential property of *anti*-symmetry, ansatz was made suitable to be used in the variational principle (to be noted, earlier in 1926, Heisenberg and Dirac [10] used the principle of *anti*-symmetry). Considering all these parallel developments, Hartree reformulated his original theory (with the inclusion of Born–Oppenheimer approximation [11]), and made it more suitable for solving time-independent Schrodinger equation, applicable to real physical systems. Later this revised theory gained the popularity as the “Hartree–Fock Method” (HF Method) [3, 12, 13].

Then it was realized that the non-inclusion of electron correlations in HF Method is one major setback and was needed to be addressed properly. This lead to the development of many well-ordered approaches, which were able to appropriately address the correlation issue associated with HF method. Approaches like, Moller–Plesset Perturbations theory (with 2^ND^ order consideration, frequently used one is the MP2 method) [14], Configuration Interaction (CI) [15], Multi-configuration Self-Consistent Field (MC-SCF) [16], Complete Active Space Self-Consistent Field (CASSCF) [17], Coupled Cluster (CC) [18–20], are the few notable theoretical developments. All these well-established theories are collectively known as the *post-HF* theories [21, 22]. Worth to mention here that, the HF theory was not only the bedrock for these post-HF theories [21, 22], but also contributed significantly for the developments of the well-known DFT (density functional theory) theory [23, 24]. Many DFT based methodologies are now being used extensively by almost all the researchers worldwide, for various areas of scientific research [25–31].

When computational capabilities were still in their emerging stage (around in the 70’s and 80’s of the 20^TH^ century), the long well-established HF theory, was in the forefront of computational chemistry research. At present, looking at the trends in the use of methodologies in computational chemistry research, one can see the general tendencies for using post-HF theories (mainly for relatively smaller molecular systems) and DFT (for medium to larger systems) [21, 22, 28, 30–34]. While the post-HF theories have the advantage of being superior to basic HF approach, in producing accurate results (many times very close to experiment), at the same time, being computationally expensive they are generally not favoured for medium to large systems. About the DFT based methodologies, current trends clearly show that they are the most widely used computational tools by all the computational chemists (being applied to small to medium to large systems, or to say all kinds of systems). With wide varieties of functionals being available, no wonder, application of DFT based methodologies became the main choice for the computational chemists in the present era of science [21, 32–34].

**Scheme 1:**
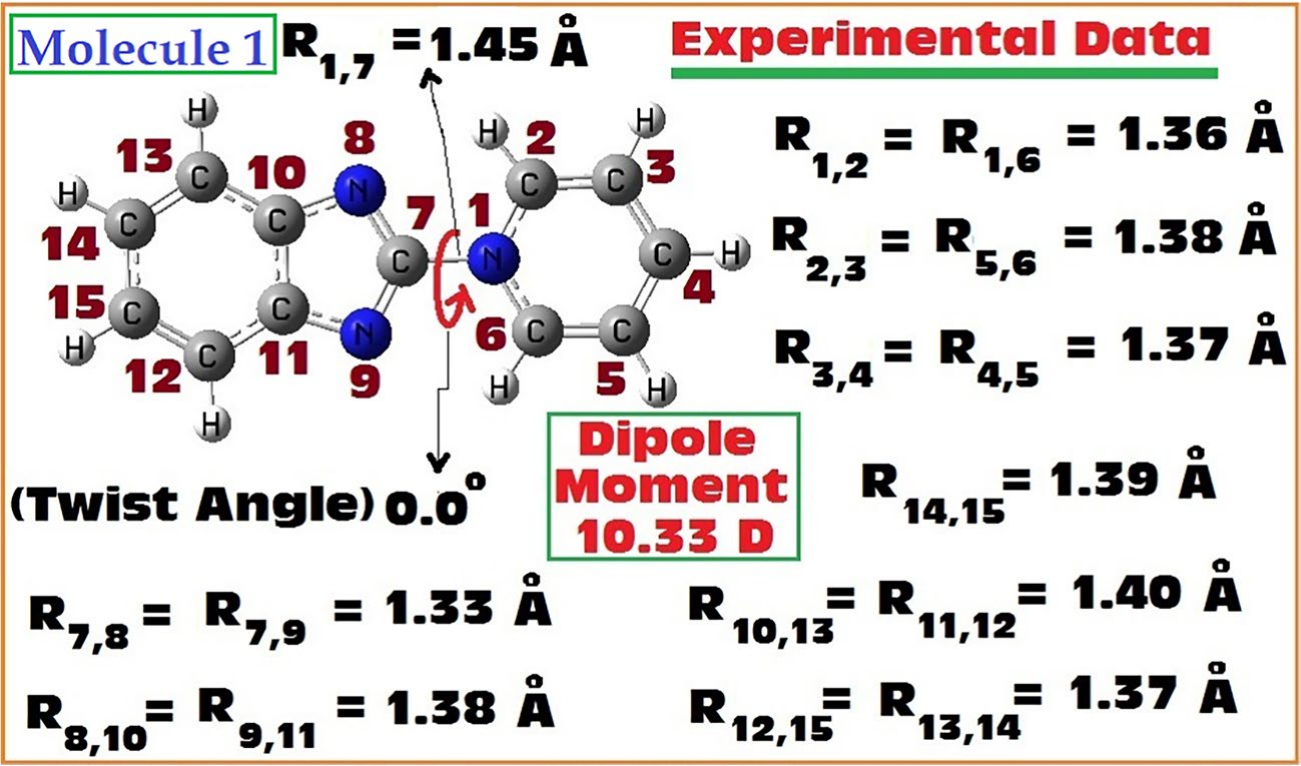
Optimized geometry of Molecule 1 shown with available experimental crystallographic data and experimental dipole moment [35, 36]

About the strong dominance of DFT in the field of organic chemistry, one can easily see that besides many profound theoretical articles, now-a-days even most of the experimental articles in organic chemistry are most often augmented with a substantial portion of DFT computations [21, 28, 30, 33]. About the HF method, now a days it is generally not the method of choice for many researchers, even in the field of computational organic chemistry. In this contribution, I investigated the structure–property correlation of some well-known zwitterionic organic molecules, which were synthesized by Boyd, long-time back in 1966 (Scheme 1) [35]. Main motivation of this study is to show that sometimes one may be able to achieve equally good or even better performances by HF method compared to DFT-based methodologies, and thus HF method can still be treated as suitable method of choice for computational organic chemistry. Then in 1987 (after 20 years), Alcalde et al. [36], resynthesized these Boyd’s zwitterions (pyridinium benzimidazolates), and investigated their crystal structures as well as one of the fundamental molecular properties, the dipole moments. Based on its relatively large dipole moment value, Molecule 1 (Scheme 1) was subjected to some interesting studies in the later years, mainly for the properties directly/indirectly linked to its dipole moment as well as its interesting charge transfer property.

For the Molecule 1, around 10 years later, Abe et al. [37] investigated it for nonlinear optics, and compared their computational results from HF method, with the experimental dipole moment value (10.33D) reported by Alcalde et al. [36]. They found that HF method was able to reproduce the dipole moment almost accurately. In another relatively recent work (13–14 years after Abe et al.) by Pawlowska et al. (also co-authored with J. Abe) [38], on the same molecule, they mentioned about the better agreement between the HF dipole moment with the experiment compared to DFT, but they were a bit unconvinced on the performance of HF method. Hence, the question arises “Are we undervaluing the results produced by HF method by not considering it seriously?” or “by effectively reproducing the experimental results for zwitterionic systems, is it showing cues for its usefulness?” Hence to find an answer to the posed questions, I carried out a detailed investigation of the some already synthesized zwitterionic molecules using a wide range of quantum mechanical methods, ranging from classical semi-empirical methods, to DFT to post-HF methods and along with the HF methods. Not only the dipole moment, but also several other fundamental properties were computed to assess the performances of various methodologies.

## Computational methods

All the computations for the molecules considered in this work were carried out using Gaussian 09 quantum chemistry program [39]. A wide range of quantum mechanical methods were used to investigate the structure, dipole moment and other fundamental properties of the molecules and are directly compared with experimental data. During the structural optimizations, no symmetry restrictions were imposed (in all the methods). Although Alcalde et al. [36] reported Molecule 1 as fully planar, optimization without symmetry restrictions will be able to inactivate any constraints to the free rotation of the two aryl rings. Besides the HF [40], many well-known DFT methodologies like, B3LYP [41, 42], B3PW91 [41, 43], TPSSh [44], BMK [45], CAM-B3LYP [46], LC-ωPBE [47], M06-2X [48], M06-HF [49], ωB97xD [50, 51], and post-HF methods like, MP2 [14], CASSCF [17], CISD [15], QCISD [52], CCSD [18–20], and semi-empirical methods like, AM1 [53], PM3MM [54], PM6 [55], Huckel [56], CNDO [57], were used in this investigation. From the computed vibrational frequencies, true local minimum with all positive frequencies or no negative eigen values in the Hessian, were established for the reference molecule in the above methodologies.

## Results and discussions

### Molecular structures

The fully optimized geometries of the Pyridinium Benzimidazolate zwitterion (Molecule 1) computed using all the methods mentioned in Sect. "Computational methods", were analysed and important structural parameters are shown in Table 1. These computed structural data were compared with the available experimental crystal structure data (provided in Scheme 1) to assess the effectiveness of various quantum mechanical methods in reproducing the experimental structure of Molecule 1. For Molecule 1, one of the important structural parameters is the twist angle between the two aryl units (donor–acceptor junction: D_2,1,7,8_). Experimental crystal structure predicted the molecule to be fully planar, with a 0.0° twist angle [36]. All the computational methods also predicted it to be fully planar like the experiment, except the MP2 method. MP2 computations predicted it to be 13.7° twisted at the junction, quite unusual behavior compared to all other methodologies. To eliminate any possibility of insufficiency of basis set consideration for MP2, I carried out full optimization with larger basis sets. Computations with aug-cc-pVTZ failed due to the insufficient computing infrastructure available in my lab. Then computation was carried out using aug-cc-pVDZ basis set.

**Table 1:** Important structural parameters (experimental crystal structure data: Scheme 1 [36]) of the Molecule 1, computed using various methodologies

Structural Parameters	R_1,2_ (R_1,6_)	R_2,3_ (R_5,6_)	R_3,4_ (R_4,5_)	R_7,8_ (R_7,9_)	R_8,10_ (R_9,11_)	R_10,13_ (R_11,12_)	R_12,15_ (R_13,14_)	R_10,11_	R_14,15_	R_1,7_	D_2,1,7,8_ (D_6,1,7,9_)
Hartree–Fock (HF) Methodology											
HF/6-31G	1.344	1.375	1.389	1.321	1.380	1.399	1.375	1.414	1.414	1.433	0.0
HF/6-31G(d,p)	1.337	1.373	1.386	1.306	1.370	1.400	1.372	1.408	1.411	1.447	0.0
HF/6–31 + + G(d,p)	1.338	1.375	1.387	1.306	1.371	1.400	1.375	1.409	1.412	1.448	0.0
HF/6–311 + + G(d,p)	1.336	1.373	1.386	1.304	1.371	1.399	1.374	1.407	1.411	1.449	0.0
Density Functional Theory (DFT) Methodologies											
B3LYP/6-31G(d,p)	1.363	1.381	1.397	1.340	1.368	1.409	1.384	1.442	1.421	1.423	0.0
B3LYP/6–311 + + G(d,p)	1.362	1.379	1.395	1.336	1.368	1.406	1.383	1.438	1.420	1.425	0.0
B3PW91/6–31 + + G(d,p)	1.359	1.381	1.396	1.336	1.366	1.407	1.384	1.438	1.420	1.420	0.0
TPSSh/6–31 + + G(d,p)	1.366	1.383	1.400	1.342	1.371	1.410	1.388	1.442	1.423	1.423	0.0
BMK/6–31 + + G(d,p)	1.354	1.388	1.401	1.335	1.369	1.414	1.389	1.440	1.428	1.427	0.0
CAM-B3LYP/6–31 + + G(d,p)	1.351	1.379	1.392	1.327	1.371	1.404	1.381	1.426	1.417	1.435	0.0
M06-2X/6–31 + + G(d,p)	1.352	1.382	1.394	1.328	1.370	1.407	1.382	1.429	1.419	1.437	0.0
M06-HF/6–31 + + G(d,p)	1.357	1.378	1.393	1.332	1.367	1.404	1.382	1.431	1.417	1.427	0.0
LC-ωPBE/6–31 + + G(d,p)	1.344	1.377	1.389	1.320	1.372	1.403	1.376	1.416	1.415	1.443	0.0
ωB97xD/6–31 + + G(d,p)	1.351	1.381	1.393	1.328	1.373	1.405	1.382	1.426	1.417	1.436	0.0
Post-Hartree–Fock (post-HF) Methodologies											
MP2/6–31 + + G(d,p)	1.360	1.387	1.397	1.347	1.374	1.411	1.387	1.439	1.423	1.428	13.7 (0.5)^**a**^
CASSCF/6–31 + + G(d,p)	1.337	1.375	1.387	1.316	1.375	1.411	1.366	1.405	1.424	1.448	0.0
CISD/6-31G	1.354	1.383	1.396	1.335	1.388	1.407	1.381	1.424	1.422	1.437	0.0
QCISD/6-31G	1.376	1.400	1.413	1.359	1.407	1.424	1.399	1.441	1.440	1.446	0.0
CCSD/6-31G	1.374	1.400	1.413	1.359	1.405	1.424	1.398	1.440	1.440	1.446	0.0
Semi-Empirical Methodologies											
PM3MM	1.380	1.390	1.392	1.398	1.369	1.421	1.365	1.442	1.429	1.405	0.0
PM6	1.383	1.395	1.398	1.400	1.368	1.428	1.367	1.473	1.442	1.418	0.0
AM1	1.374	1.399	1.396	1.413	1.364	1.423	1.368	1.481	1.426	1.420	0.0
Huckel	1.358	1.377	1.385	1.349	1.385	1.400	1.376	1.430	1.397	1.434	0.0
CNDO	1.358	1.377	1.385	1.349	1.385	1.400	1.376	1.430	1.397	1.434	0.0

^**a**^ represents the value from MP2/aug-cc-pVDZ basis set optimization

Interestingly, the obtained optimized structure was found to be almost fully planar like other methods (other structural parameters obtained using this large basis set were found to be same or close to the values obtained using 6–31 +  + G(d,p) basis set). A reduction of twist angle from 13.7° to 0.5°, with the change of basis set from 6–31 +  + G(d,p) to aug-cc-pVDZ clearly indicates that, with cc-pVTZ basis set it will possibly show a fully planar conformation as predicted by all other methods. Thus, one can say that while dealing with biaryl types of zwitterionic molecules which are susceptible for internal rotations at the inter-ring junctions, with MP2 methodology a large basis set is essential to address the structures more accurately [58]. As other methods (even the well-known HF theory) were found to be more suitable in addressing the structural aspects, even in the lower basis set domains, hence MP2 method is not advisable for similar zwitterionic systems for researchers, who are limited with computing resources.

Like the inter-ring twist angle, another important structural parameter is the junction bond (R_1,7_: Scheme 1) and the reported experimental value is 1.45 Å. Analysis of the computed values from various methodologies (Table 1) it can be observed, while all the DFT methods predicted underestimated values (except LC-ωPBE) compared to experiment [36], the post-SCF (CASSCF, CCSD, QCISD) methods (except MP2) along with the HF methods (with different basis sets) predicted values very much closer (same cases exact values) to the experiment [36]. Interesting to note here that almost exact matching values with the experiment were shown by HF methods with larger basis sets. This is again a clear indication that, even simple HF method is quite suitable in reproducing the experimental parameters. Other structural parameters shown in Table 1 are the directly associated with the two aryl units present in the molecule, and observations indicate the DFT based methods were reproducing some of those values closer to the experimental data, compared to all other methods (even the post-SCF methods). Nevertheless, the central aryl-aryl junction bond and the twist angle which are important in establishing effective communication between the donor and acceptor sides, indicated that the values obtained in HF and post-HF methods are closer to experimental results than the DFT based methods. A direct comparison of CASSCF/6–31 +  + G(d,p) and HF/6–31 +  + G(d,p) methods can be made as both were performed in same basis set combinations. Such a comparison indicates that both the methodologies predicted near equivalent geometries, and very close to the experimental crystal structure data [36]. Based on this analogy and comparison with the experimental data, one can say that even HF/6–31 +  + G(d,p) is the most efficient methodology to properly account for the structure of this zwitterion and needed to be tested for other zwitterionic systems to see if it is a general trend or not.

### Dipole moments

Density functional approaches are well-known to be predicting improved energies and are generally believed that they can inevitably be more accurate in predicting electron densities compared to Hartree–Fock method. This belief has been questioned by researchers in the recent times [59–68]. It is well-known that one of the simple molecular properties is the dipole moment of any molecule, and often it is used to precisely understand the electron density distributions of any polar molecule. Hence, to assess how various well-known quantum mechanical methods predict the molecular dipole moment (indirect inferences can be obtained about the performances of various methodologies on predictions of appropriateness of the distributions of electron densities) is the main intention of this analysis [59]. To test this aspect for the first time, for a zwitterionic molecule, both the crystal structure data and the experimental dipole moment were available for one such molecule (Molecule 1, Scheme 1). Another molecule for which both the crystal structure and dipole moment were also reported in the same work but owing to its exceedingly large size (multiple phenyl and alkyl substituted derivative of the reference molecule), such an investigation (with all the methods investigated in this work) will be impossible for us (with our limited computing resources) [36].

Computed dipole moment data for the Molecule 1, obtained from various methodologies are shown in Table 2. As can be seen from Table 2, when compared with the experimental dipole moment of 10.33 D (in the same report, Alcalde et al. also computed the dipole moment with semi-empirical method and reported the value as 11.06 D [36]), it can be seen that, while the post-HF and HF methods predicted the dipole moment values closer to the experimental values, at the same time the predicted values by the hybrid DFT and also the semi-empirical methods were found to be deviated strongly from the experimental value. To be noted that most of the long-range corrected DFT methods predicted the dipole moment values closer to experimental value. Worth to mention here that, while HF and post-SCF methods predicted either nearly equivalent or slightly overestimated values compared to the experiment, at the same time the long-range corrected methods predicted slightly underestimated values (even the other DFT and semi-empirical methods predicted more underestimated deviations from the experimental dipole moment).

**Table 2:** Dipole moments in Debye (D) for the Molecule 1, computed using various methodologies. Experimental dipole moment shown is from reference [36]

Methods	μ_T_ (= μ_X_)	Methods	μ_T_ (= μ_X_)
HF/6-31G	10.31	LC-ωPBE/6–31 + + G(d,p)	9.90
HF/6-31G(d,p)	10.50	ωB97xD/6–31 + + G(d,p)	9.43
HF/6–31 + + G(d,p)	10.72	CASSCF/6–31 + + G(d,p)	10.40
HF/6–311 + + G(d,p)	10.73	CISD/6-31G	10.34
B3LYP/6-31G(d,p)	7.70	QCISD/6-31G	10.46
B3LYP/6–311 + + G(d,p)	7.99	CCSD/6-31G	10.45
B3PW91/6–31 + + G(d,p)	7.96	MP2/6–31 + + G(d,p)	10.80
TPSSh/6–31 + + G(d,p)	7.59	MP2/aug-cc-pVDZ	10.70
BMK/6–31 + + G(d,p)	8.82	PM3MM	7.71
CAM-B3LYP/6–31 + + G(d,p)	9.31	PM6	7.75
M06-2X/6–31 + + G(d,p)	9.19	AM1	8.65
M06-HF/6–31 + + G(d,p)	8.36	Experimental: μ = 10.33 Debye	

Dipole moment of 10.31 D predicted by simple HF/6-31G was found to be almost same as reported in earlier experimental value of 10.33 D [36]. With other basis sets, the HF method predicted slightly larger values compared to experiment. At the same time a value of 10.34 D predicted by post-HF method CISD with 6-31G basis set can be regarded as exact match with the experiment. Other post-SCF methods predicted the dipole moment values slightly higher than experiment. All the long-range corrected DFT methods, predicted the dipole moments comparatively closer to experimental value compared to all the hybrid DFT methods, but still larger differences compared to experiment were observed. This gives a clear indication that HF method is quite suitable in predicting not only the structure and but also dipole moment of the zwitterionic molecule (Molecule 1) more efficiently than all the DFT based methods. Hence, at this stage one can say that even the simple HF method can be considered as suitable in accounting the electron density distribution around of the zwitterionic Molecule 1 and can also be expected to efficient in the cases of other similar zwitterionic systems (definitely need more investigations to validate this claim).

To test the observed suitability of HF, we looked in the same work of Alcalde et al. [36], we found three more molecules for which they reported the experimental dipole moments, hence they can be ideal candidates for the test. Out of three molecules, two molecules are extremely large (with multiple phenyl substitutions) and hence it will not be possible for us to do the line of investigations carried out with those two molecules. Luckily one molecule was of reasonable size and a substituted derivative of Molecule 1. The methyl substituted derivative of Molecule 1 (pyridinium benzimidazolate), which is Molecule 2 is shown in Table 3 (geometry of molecule is shown in its optimized orientation). Computed dipole moments from various methodologies are also shown in Table 3.

**Table 3:**
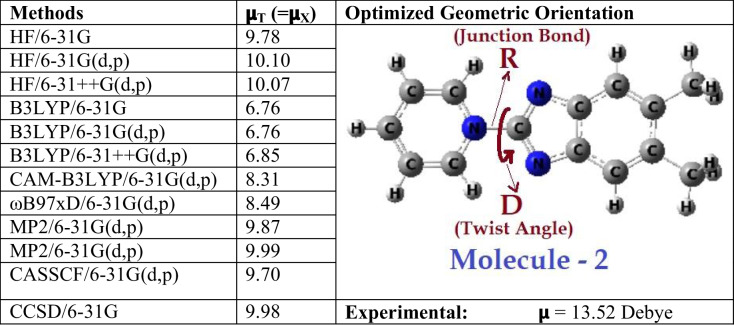
Dipole moments in Debye (D) for the Molecule 2, computed using various methodologies. Experimental dipole moment shown is from reference [36]. Molecule 2 in its optimized geometric orientation is also shown

Though experimental dipole moment for Molecule 2 was reported, but Alcalde et al. [36], did not report the crystal structure data for it. But, in view of similarity in structure and moreover as the two methyl substituents are remotely located from the bridge, hence we can expect Molecule 2 be structurally very close to Molecule 1. For all the investigated methodologies, we found the Molecule 2 to be in fully planar conformations (even in MP2 with smaller basis sets). Now about the central junction bond, we observed slightly different values in various methodologies compared to Molecule 1 (it was 1.45 Å for Molecule 1). While HF and post-HF methodologies predicted it to be closer to 1.45 Å (HF 1.43–1.45 Å, CASSCF: 1.45 Å, CCSD: 1.44 Å), other methodologies predicted slightly underestimated values (B3LYP: 1.42–1.43 Å, CAM-B3LYP: 1.43 Å, MP2:1.42–1.43 Å, ωB97xD: 1.43 Å).

Now about the dipole moments, unlike Molecule 1, here for Molecule 2 surprisingly we observed that in all the methodologies predicted underestimated values of dipole moments compared to experimental value of 13.52 D. B3LYP predicted a value of 6.85 D which is almost half of the experimental value. But with the long-range corrected methodologies, like CAM-B3LYP and ωB97xD, we observed improved values than the B3LYP. Among all the methodologies, HF predicted the largest dipole moment (around 10.1 D). Slightly lower values of observed in all the post-HF methodologies, compared to the HF predicted dipole. These marginal differences may be due to the lower level of basis sets employed for most of the post-HF methodologies. Despite the underestimated values in all methodologies, once again HF was proved itself to be more suitable by predicting close to the experiment the experimental value (only a difference of 3.4 D was observed). Based on the similarity of trends shown by both Molecules 1 and 2, with respect to the performances, we can say that among various methodologies, simple HF methodology is quite useful in predicting structure–property correlations closer to experiment. Though at this stage exclusively we can’t advocate the persistence of such a trend for all kind of zwitterions but based on the good performances shown by HF methodology and unusual worst performance by B3LYP for the current zwitterions, we can say that one need to be careful while computationally dealing with zwitterions.

### Frontier molecular orbital energies

Besides the observations of the dipole moment values, one of the key molecular properties which is going to be directly affected by the variances in distributions of electron densities (shown by various methodologies) is the energies related to frontier molecular orbitals. Hence frontier molecular energetics data for the Molecule 1, computed using various methodologies were tabulated and are shown in Table 4. As expected, large differences were observed in the energetics of HOMO (highest occupied molecular orbital: E_HOMO_), LUMO (lowest unoccupied molecular orbital: E_LUMO_) and the ΔE_HLG_ (energy difference between the two: E_LUMO_—E_LUMO_).

From Table 4, it can be seen that the lowest values of ΔE_HLG_ were obtained for the hybrid DFT based methods and larger HOMO–LUMO gaps were observed for the HF method, with nearly similar values for the post-HF methods. At the same time all the long-range corrected DFT methods showed larger HOMO–LUMO gaps compared to hybrid DFT methods and lower gaps compared to HF as well as post-HF methods (except M06-HF which predicted it to be like the hybrid DFT methods). As orbitals play primary roles in charge transfer and excitation properties of a molecule, hence such a situation can be expected to affect many related properties. Based on the near similar values of HF and post-SCF methods, and at the same time approaching values for larger HLG values for the long-range corrected methods, one can say that the hybrid DFT methods may not be properly accounting the frontier molecular orbital energetics for this molecule.

**Table 4:** Orbital energies of HOMO, LUMO and HOMO–LUMO gap in electron Volts (eV) of the Molecule 1 computed using various methodologies ^**a**^ represents the values from aug-cc-pVDZ basis set computation

Methods	E_HOMO_	E_LUMO_	ΔE_HLG_	Methods	E_HOMO_	E_LUMO_	ΔE_HLG_
HF/6-31G	-7.03	0.70	7.73	CAM-B3LYP/6–31 + + G(d,p)	-6.67	-1.86	4.81
HF/6-31G(d,p)	-6.85	0.85	7.70	M06-2X/6–31 + + G(d,p)	-6.59	-2.12	4.47
HF/6–31 + + G(d,p)	-7.10	0.38	7.48	M06-HF/6–31 + + G(d,p)	-5.71	-2.81	2.90
HF/6–311 + + G(d,p)	-7.12	0.36	7.48	LC-ωPBE/6–31 + + G(d,p)	-7.85	-0.95	6.90
B3LYP/6-31G(d,p)	-5.12	-2.58	2.54	ωB97xD/6–31 + + G(d,p)	-7.21	-1.25	5.96
B3LYP/6–311 + + G(d,p)	-5.51	-3.00	2.51	MP2/6–31 + + G(d,p)	-6.93 (-6.86)^**a**^	0.20 (0.15)^**a**^	7.13 (7.01)^**a**^
B3PW91/6–31 + + G(d,p)	-5.52	-2.95	2.57	CISD/6-31G	-7.04	0.63	7.67
TPSSh/6–31 + + G(d,p)	-5.13	-3.07	2.06	QCISD/6-31G	-7.02	0.48	7.50
BMK/6–31 + + G(d,p)	-6.10	-2.24	3.86	CCSD/6-31G	-7.03	0.49	7.52

Analysis of the trends in the stabilities of the frontier molecular orbitals indicate that while the HF and post-SCF methods predicted the HOMO to be stabilized, at the same time the DFT based methods predicted it to be less stabilized (whereas LC-ωPBE and ωB97xD methods stabilized HOMO like that of the HF method). Interesting observations were found for the energetics of LUMO. While all the post-HF and HF methods predicted a destabilized LUMO (positive energies), all the DFT based methods predicted it to be highly stabilized with negative energy values. Such a situation can be attributed as being largely responsible for the observed molecular band gap (of HOMO–LUMO gap) for different methodologies. This gives a clear indication that there might be some intrinsic problems associated with the methodologies, which ultimately affects the electron density distributions around the molecule, and consequently the orbital energies as well as the dipole moment values are also getting strongly influenced. Many recent works proposes that such possibilities may be analysed in the light of the localization-delocalization problem [59–61, 65, 66].

### Localization and delocalization problems

As discussed previously, the question arises here is that the observed differences in properties can exclusively attributed only to the difference in geometries or some other factors might be affecting these behaviours shown by HF and DFT methods. To test this, I took the optimized geometry of the B3LYP/6–311 +  + G(d,p) and computed the dipole moment at HF/6–311 +  + G(d,p) level, without doing any further optimizations. Also, to avoid any basis set related adverse effects, I investigated the above situation with a 6–311 +  + G(d,p) basis set. Interestingly, the observed dipole moment was found to be 10.85 D, larger than the values obtained from B3LYP/6–311 +  + G(d,p) full optimization, and slightly larger than HF/6–311 +  + G(d,p) full optimization. This is clear indication that the geometry may not have significant influence on the observed dipole moment. Also, influence of the geometry only, on the dipole moment can be ruled out based on the MP2 results, where it showed a completely different geometry (with a twisted conformation around the aryl-aryl junction) compared to all other methods, but a dipole moment was found to be close to experimental value. Interestingly according to S. Nam et al., [69], “*Problematic calculations are density-sensitive and using HF densities fixes these issues*.” Not only that, but also all the other post-HF approaches produced very similar geometry and dipole moment values like that the traditional HF approach.

**Fig. 1:**
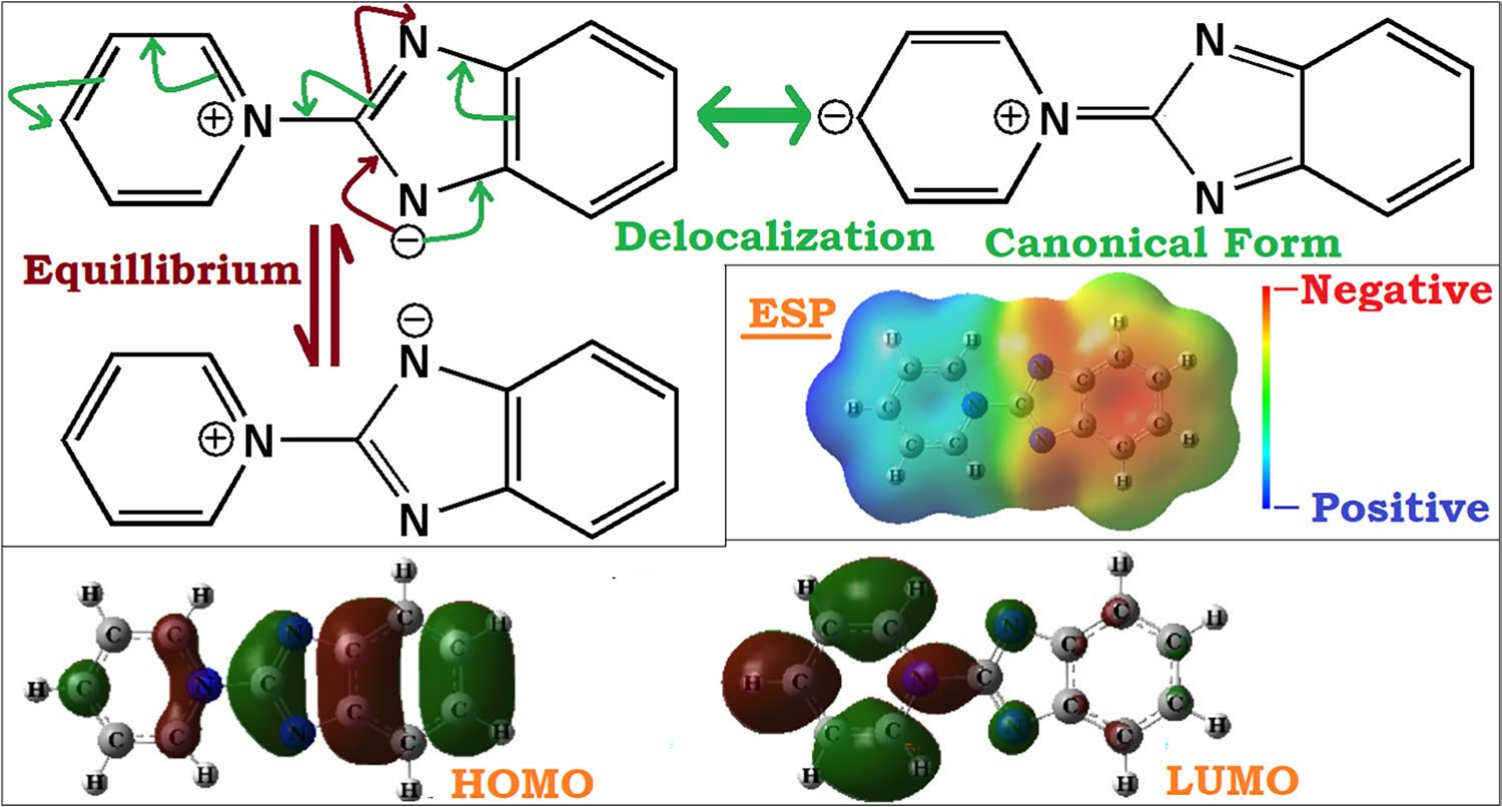
Pyridinium benzimidazolate zwitterion (Molecule 1) in its possible canonical forms, frontier molecular orbitals (HOMO & LUMO), and ESP map. Diagrams are not to scale

Based on all these observations, I tried to move our focus on the nature of problems associated with the HF and DFT. Computed electrostatic potential (ESP) map, frontier molecule orbitals (HOMO & LUMO) and canonical forms of the reference molecule (Fig. 1) are used in the analysis of the localization and delocalization issues associated with the HF and DFT methodologies. One of the problems described in many earlier works [59–75], and recently highlighted in a perspective article by H. J. Kulik, is the localization/delocalization problem associated with the HF and DFT methodologies. H. J. Kulik stated, “*Degree of localization or delocalization is a problem in both approximate density function theory and in Hartree–Fock.*” [70]. The perspective also highlights the pernicious nature of this problem, like, how sometimes over-delocalization (DFT) can turn an insulator to metal and over-localization (HF) can produce wrong results in barrier height estimations or energetics of a reaction [70]. In the present context, the reference molecule being zwitterionic nature, this problem looks more probable, as a slightest over- or under-estimation of the localization/delocalization can strongly affect the structure–property correlations. A delocalization may impact some stabilization to a particular canonical form (Fig. 1) and may ultimately affect the frontier molecule orbital energies. Figure 1 shows that both HOMO & LUMO are exhibiting localized natures of population density distributions, hence any delocalization (representative canonical form) may affect the density distributions as well as the energetics. Also, the ESP map clearly indicates centralized regions of positive and negative potentials in the molecule (Fig. 1) and any delocalization can directly affect not only the potential distributions of the consolidated regions, but also many other directly related properties of the molecule.

Associated to delocalization problem, self-interaction error associated with DFT is discussed in a recent work of Lee et al. [71]. They discussed about the difference in properties predicted by DFT and HF method for anions. They even suggested that HF theory can be a naive solution to address the problem, shown by DFT methods in predicting the frontier molecular orbital energies and other related properties of anionic systems [71]. As the systems considered here are zwitterionic natures (with the donor part anionic in nature), hence underperformance of DFT compared to HF may also be addressed as explained by Lee et al. [71]. In the present case I have also observed very similar problems shown by the DFT methods in predicting the frontier molecular orbital energies of the reference zwitterionic molecule (Table 4 and related discussions in Sect. "Frontier molecular orbital energies"). As discussed in the beginning of this section, the orbital energies E_HOMO_ = -5.51 eV and E_LUMO_ = -3.00 eV shown by the B3LYP/6–311 +  + G(d,p), got changed to -6.93 eV and + 0.14 eV respectively when computed at HF/6–311 +  + G(d,p) level, without doing any further optimization of the geometry obtained at B3LYP/6–311 +  + G(d,p) method (these results are similar and close to the results obtained from the HF/6–311 +  + G(d,p) full optimizations). This clearly indicates that the localization/delocalization problem associated with the HF and DFT methods plays very significant role. In the case of DFT methods, it was observed that the associated delocalization issue gave a stabilized LUMO (negative energy) and a slightly destabilized HOMO, in all the cases compared to HF method. Thus, the localization issue associated with the HF method was found to be in an advantageous position to accurately address the orbital energetics. This inference can be drawn based on the near equivalent results produced by the post-HF theories. Also, to mention here that DFT methods incorporating the long-range interaction factors and dispersion corrections were found to be in better positions in producing the orbital energetics (still the LUMO energies were found to be negative) and consequently structure and dipole moments were also found to be close to the HF results (LC-ωPBE was found to be performing better than all other methods). Other inference can be drawn is that localization issue associated with HF method showing a preference towards the more zwitterionic behaviour of the molecule, and at the same time the delocalization issue associated with the DFT based methods showing the preferences for a more quinonoid type behaviour for the reference molecule.

## Conclusions

This contribution reports the computational results for Boyd’s zwitterions (pyridinium benzimidazolates) and compares them with the experimental structural data and properties already reported in the literature. Computational investigations were carried out using various methodologies like, HF, B3LYP, CAM-B3LYP, BMK, B3PW91, TPSSh, LC-ωPBE, M06-2X, M06-HF, ωB97xD, MP2, CASSCF, CCSD, QCISD, CISD, Huckel, CNDO, AM1, PM3MM and PM6, to evaluate the performances of all these well-known computational methods, against the available experimental structural and dipole moment data. A comparison with the experimental data clearly indicates that HF and post-HF methodologies were able to reproduce the experimental data (both the structure and dipole moments). At the same time Hybrid DFT methodologies showed substantial deviations from the experimental data. But, with the inclusion of long-range interaction corrections and/or dispersion corrections in DFT, some improved performances in reproducing the experimental data were observed. Better performances of HF and post-HF methodologies compared to DFT, were explained in the light of localization/delocalization problem associated with the HF and DFT. It was observed that localization issue associated with HF preferring more zwitterionic, and the delocalization issue associated with the DFT based methods, showing the preferences for a more quinonoid type behaviour for the pyridinium benzimidazolate type of zwitterions. These molecules being zwitterionic nature, a slightest over- or under-estimation of the localization/delocalization can strongly affect the structure–property correlations.

## Data Availability

This article doesn’t contain any supporting information as most of the important data are already provided in the manuscript. However, optimized coordinates of the molecule for various methodologies can be obtained from the author, through email request.

## References

[1] Hartree DR (1928). The Wave Mechanics of an Atom with a Non-Coulomb Central Field. Math Proc Camb Philos Soc.

[2] Schrödinger E (1926). An Undulatory Theory of the Mechanics of Atoms and Molecules. Phys Rev.

[3] Shankar R (1994) Principles of Quantum Mechanics (2nd Ed.), Kluwer Academic/Plenum Publishers

[4] Slater JC (1928). The Self Consistent Field and the Structure of Atoms. Phys Rev.

[5] Gaunt JAA (1928). Theory of Hartree's Atomic Fields. Math Proc Camb Philos Soc.

[6] Slater JC (1930). Note on Hartree's Method. Phys Rev.

[7] Fock VA (1930). Näherungsmethode zur Lösung des quantenmechanischen Mehrkörperproblems. Z Phys.

[8] Fock VA (1930). Selfconsistent field mit Austausch für Natrium. Z Phys.

[9] Slater JC (1951). A Simplification of the Hartree-Fock Method. Phys Rev.

[10] Dirac PAM (1930). The Principles of Quantum Mechanics.

[11] Born M, Oppenheimer JR (1927). Zur Quantentheorie der Molekelm. Annalen der physic.

[12] Levine IN (1991) Quantum Chemistry (4th Ed.), Englewood Cliffs, New Jersey: Prentice Hall

[13] Szabo A, Ostlund NS (1961) Modern Quantum Chemistry, Mineola, New York: Dover Publishing

[14] Møller C, Plesset MS (1934). Note on an approximation treatment for many-electron systems. Phys Rev.

[15] Pople JA, Seeger R, Krishnan R (1977). Variational Configuration Interaction Methods and Comparison with Perturbation Theory. Int J Quantum Chem.

[16] Eade RHA, Robb MA (1981). Direct minimization in MC SCF theory - the Quasi-Newton method. Chem Phys Lett.

[17] Klene M, Robb MA, Frisch MJ, Celani P (2000). Parallel implementation of the CI-vector evaluation in full CI/CAS-SCF. J Chem Phys.

[18] Purvis GD, Bartlett RJ (1982). A full coupled-cluster singles and doubles model - the inclusion of disconnected triples. J Chem Phys.

[19] Krishnan R, Schlegel HB, Binkley JS, People JA (1978). Electron Correlation Theories and Their Application to the Study of Simple Reaction Potential Surfaces. Int J Quantum Chem.

[20] Bartlett RJ, Purvis GD (1978). Many-body perturbation-theory, coupled-pair many-electron theory, and importance of quadruple excitations for correlation problem. Int J Quantum Chem.

[21] Cramer CJ (2002) Essentials of Computational Chemistry, Chichester: John Wiley & Sons, Ltd

[22] Magnasco V (2009) Post-Hartree-Fock Methods, Methods of Molecular Quantum Mechanics, John Wiley and Sons

[23] Kohn W, Sham LJ (1965). Self-Consistent Equations Including Exchange and Correlation Effects. Phys Rev.

[24] Hohenberg P, Kohn W (1964). Inhomogeneous electron gas. Phys Rev.

[25] Seminario JM (Eds) (1966) Recent Developments and Applications of Modern Density Functional Theory (1st Ed.), Elsevier Science

[26] Bagayoko D (2014). Understanding density functional theory (DFT) and completing it in practice. AIP Adv.

[27] Burke K, Wagner LO (2014). DFT in a nutshell. Int J Quant Chem.

[28] Becke AD (2014). Perspective: Fifty years of density-functional theory in chemical physics. J Chem Phys.

[29] Parr RG, Yang W (1989). Density-Functional Theory of Atoms and Molecules.

[30] Burke K, Werschnik J, Gross EKU (2005). Time-dependent density functional theory: Past, present, and future. J Chem Phys.

[31] Teale AM (2022). DFT Exchange: Sharing Perspectives on the Workhorse of Quantum Chemistry and Materials Science. Phys Chem Chem Phys.

[32] Verma P, Truhlar GG (2020). Status and Challenges of Density Functional Theory. Trends Chem.

[33] Cohen AJ, Mori-Sanchez P, Yang W (2012). Challenges for density functional theory. Chem Rev.

[34] Sim E, Song S, Vuckovic S, Burke K (2022). Improving Results by Improving Densities: Density-Corrected Density Functional Theory. J Am Chem Soc.

[35] Boyd GV (1966). An aza-analogue of N-phyridinium cyclopentadienide. Tet Lett.

[36] Alcalde E, Dinares I, Elguero J, Fayet JP, Vertut MC, Miravitlles C, Molins E (1987). Azinium azolate inner salts: synthesis and structural studies. J Org Chem.

[37] Abe J, Shirai Y, Nemoto N, Miyata F, Nagase Y (1997). Heterocyclic Pyridinium Betaines, A New Class of Second-Order Nonlinear Optical Materials: Combined Theoretical and Experimental Investigation of First-Order Hyperpolarizability through ab Initio, INDO/S, and Hyper-Rayleigh Scattering. J Phys Chem.

[38] Pawlowska Z, Lietard A, Aloïse S, Sliwa M, Idrissi A, Poizat O, Buntinx G, Delbaere S, Perrier A, Maurel F, Jacques P, Abe J (2011). The excited state dipole moments of betaine pyridinium investigated by an innovative solvatochromic analysis and TDDFT calculations. Phys Chem Chem Phys.

[39] Frisch MJ (2009). Gaussian 09.

[40] Roothaan CCJ (1951). New Developments in Molecular Orbital Theory. Rev Mod Phys.

[41] Becke AD (1993). Density-functional thermochemistry. III. The role of exact exchange. J Chem Phys.

[42] Lee C, Yang W, Parr RG (1988). Development of the Colle-Salvetti correlation-energy formula into a functional of the electron density. Phys Rev B.

[43] Perdew JP, Wang Y (1992). Accurate and Simple Analytic Representation of the Electron Gas Correlation Energy. Phys Rev B.

[44] Tao JM, Perdew JP, Staroverov VN, Scuseria GE (2003). Climbing the density functional ladder: Nonempirical meta-generalized gradient approximation designed for molecules and solids. Phys Rev Lett.

[45] Boese AD, Martin JML (2004). Development of Density Functionals for Thermochemical Kinetics. J Chem Phys.

[46] Yanai T, Tew D, Handy NA (2004). New hybrid exchange-correlation functional using the Coulomb-attenuating method (CAM-B3LYP). Chem Phys Lett.

[47] Tawada Y, Tsuneda T, Yanagisawa S, Yanai T, Hirao K (2004). A long-range-corrected time-dependent density functional theory. J Chem Phys.

[48] Zhao Y, Truhlar DG (2006). Comparative DFT study of van der Waals complexes: Rare-gas dimers, alkaline-earth dimers, zinc dimer, and zinc-rare-gas dimers. J Phys Chem.

[49] Zhao Y, Truhlar DG (2008). The M06 suite of density functionals for main group thermochemistry, thermochemical kinetics, noncovalent interactions, excited states, and transition elements: two new functionals and systematic testing of four M06-class functionals and 12 other functionals. Theor Chem Acc.

[50] Chai J-D, Head-Gordon M (2008). Systematic optimization of long-range corrected hybrid density functionals. J Chem Phys.

[51] Chai J-D, Head-Gordon M (2008). Long-range corrected hybrid density functionals with damped atom-atom dispersion corrections. Phys Chem Chem Phys.

[52] Gauss J, Cremer D (1988). Analytical evaluation of energy gradients in quadratic configuration-interaction theory. Chem Phys Lett.

[53] Dewar MJS, Zoebisch EG, Healy EF (1985). AM1: A New General Purpose Quantum Mechanical Molecular Model. J Am Chem Soc.

[54] Anders E, Koch R, Freunscht P (1993). Optimization and application of lithium parameters for PM3. J Comp Chem.

[55] Stewart JJP (2007). Optimization of parameters for semiempirical methods. V. Modification of NDDO approximations and application to 70 elements. J Mol Model.

[56] Hoffmann R (1963). An Extended Huckel Theory. I. Hydrocarbons. J Chem Phys.

[57] Segal G, Pople J (1966). Approximate self-consistent molecular orbital theory. 3. CNDO results for AB2 and AB3 systems. J Chem Phys.

[58] Sitha S (2022). Planar in Brooker's Mode and Twisted in Reichardt's Mode: Defying the Steric Forces in Biphenyl Types of Zwitterionic Systems Through Metameric Resonance Stabilizations. Phys Chem Chem Phys.

[59] Hait D, Head-Gordon M (2018). How Accurate Is Density Functional Theory at Predicting Dipole Moments? An Assessment Using a New Database of 200 Benchmark Values. J Chem Theory Comput.

[60] Graziano G (2017). Quantum chemistry: DFT’s midlife crisis. Nat Rev Chem.

[61] Korth M (2017). Density Functional Theory: Not Quite the Right Answer for the Right Reason Yet. Angew Chem Int Ed.

[62] Medvedev MG, Bushmarinov IS, Sun J, Perdew JP, Lyssenko KA (2017). Density functional theory is straying from the path toward the exact functional. Science.

[63] Brorsen KR, Yang Y, Pak MV, Hammes-Schiffer S (2017). Is the Accuracy of Density Functional Theory for Atomization Energies and Densities in Bonding Regions Correlated?. J Phys Chem Lett.

[64] Hammes-Schiffer S (2017). A conundrum for density functional theory. Science.

[65] Cohen AJ, Mori-Sanchez P, Yang W (2008). Insights into current limitations of density functional theory. Science.

[66] Pribram-Jones A, Gross DA, Burke K (2015). DFT: A theory full of holes?. Ann Rev Phys Chem.

[67] Ruzsinszky A, Perdew JP (2011). Twelve outstanding problems in ground-state density functional theory: a bouquet of puzzles. Comput Theor Chem.

[68] Bryenton KR, Adeleke AA, Dale SG, Johnson ER (2023). Delocalization error: The greatest outstanding challenge in density-functional theory. WIREs Comput Mol Sci.

[69] Nam S, Cho E, Sim E, Burke K (2021). Explaining and Fixing DFT Failures for Torsional Barriers. J Phys Chem Lett.

[70] Kulik HJ (2015). Perspective: Treating electron over-delocalization with the DFT+U method. J Chem Phys.

[71] Lee GY, Bay KL, Houk K (2019). Evaluation of DFT Methods and Implicit Solvation Models for Anion-Binding Host-Guest Systems. Helv Chim Acta.

[72] Gould T, Pittalis S (2017). Hartree and exchange in ensemble density functional theory: avoiding the nonuniqueness disaster. Phys Rev Lett.

[73] Cai ZL, Sendt K, Reimers JR (2002). Failure of density-functional theory and time-dependent density-functional theory for large extended *π* systems. J Chem Phys.

[74] Jacquemin D, Femenias A, Chermette H, Ciofini I, Adamo C, André JM (2006). Assessment of several hybrid DFT functionals for the evaluation of bond length alternation of increasingly long oligomers. J Phys Chem A.

[75] Mori-Sánchez P, Cohen AJ, Yang W (2008). Localization and delocalization errors in density functional theory and implications for band-gap prediction. Phys Rev Lett.

